# Dynamic Precision Oncology for Real-Time Molecular Monitoring and Management in Urothelial Carcinoma

**DOI:** 10.3390/ijms27083474

**Published:** 2026-04-13

**Authors:** Whi-An Kwon, Yeon Jee Lee, Yong Sang Song

**Affiliations:** 1 Department of Urology, Hanyang University College of Medicine, Myongji Hospital, Goyang 10475, Republic of Korea; 2 Research Institute of Precision Medicine and Geroscience, Myongji Medical Foundation, Goyang 10475, Republic of Korea; 3 Department of Obstetrics and Gynecology, Hanyang University College of Medicine, Myongji Hospital, Goyang 10475, Republic of Korea; yjlee7283@gmail.com (Y.J.L.); yssong@snu.ac.kr (Y.S.S.)

**Keywords:** circulating tumor DNA, urothelial carcinoma, molecular residual disease, liquid biopsy, dynamic precision oncology, fibroblast growth factor receptor

## Abstract

The management of urothelial carcinoma (UC) is undergoing a paradigm shift from static anatomical staging to molecularly guided dynamic approaches that integrate time as a critical therapeutic variable. This evolution is driven by liquid biopsies, particularly circulating tumor DNA, which allow real-time tumor interrogation. We conducted this expert review to synthesize landmark evidence, enabling technologies, and implementation challenges in dynamic precision oncology for UC. In this non-systematic narrative review, we searched PubMed/MEDLINE, Embase, Web of Science, and the Cochrane Library for articles published between January 2015 and February 2026. Studies were selected based on their relevance to dynamic precision oncology, clinical actionability, and translational implementation, prioritizing landmark randomized controlled trials providing level 1–2 evidence, large prospective cohorts, and key translational studies. Enfortumab vedotin plus pembrolizumab established the new first-line standard for metastatic UC, achieving a median overall survival of 33.8 months versus 15.9 months (hazard ratio [HR] 0.51, 95% confidence interval 0.43–0.61). Circulating tumor DNA demonstrates robust prognostic value for molecular residual disease (MRD) detection (Level 2a evidence), stratifying recurrence risk with hazard ratios of approximately 4.5. Critically, the IMvigor011 trial has now provided Level 1b evidence that ctDNA-guided adjuvant atezolizumab improves both disease-free survival (DFS) (HR 0.64, *p* = 0.0047) and OS (HR 0.59, *p* = 0.0131) in ctDNA(+) patients, while validating treatment de-escalation in ctDNA(−) patients (1-year DFS 95%). Erdafitinib in patients harboring *FGFR2/3* alterations (HR 0.64) confirms the value of genomic profiling. Major limitations include the inherent selection bias of this non-systematic approach, substantial platform heterogeneity, and lack of standardization. In conclusion, dynamic precision oncology has transformed UC management, with the IMvigor011 trial establishing ctDNA-guided MRD status as the first phase 3-validated predictive biomarker framework for adjuvant therapy selection in a solid tumor. Implementation requires adherence to established standardization frameworks, cross-platform and cross-agent validations, and tiered implementation strategies to ensure equitable access across diverse resource settings.

## 1. Introduction

The clinical management of urothelial carcinoma (UC) has long relied on a rigorously defined framework structured around anatomical staging, with a critical distinction between non-muscle-invasive and muscle-invasive disease, and therapeutic strategies dictated by diagnostic tissue biopsy findings [1]. Although this anatomically based paradigm has served as the foundation of care for decades, its inherent limitations are underscored by persistently high variability in patient outcomes. Despite aggressive, curative-intent multimodal therapy, approximately 50% of patients with muscle-invasive bladder cancer (MIBC) experience disease recurrence and, ultimately, fatal outcomes. This variability reflects a fundamental limitation of the static paradigm [2,3], whereby single biopsies cannot capture the spatial and temporal heterogeneity that characterizes UC, as it overlooks the molecular complexity within primary tumors and between metastatic sites, and is unable to effectively monitor tumor evolution under therapeutic pressure [4].

This review introduces the concept of “Dynamic Precision Oncology (DPO),” a paradigm that extends traditional precision medicine by explicitly incorporating time as a critical therapeutic variable. We define DPO as a treatment paradigm that tracks intratumoral heterogeneity and evolutionary dynamics through serial molecular assessments, adapts therapeutic strategies based on real-time molecular changes, and proactively plans for future resistance development. This approach contrasts with conventional precision medicine that matches therapies to static consensus molecular patterns [5,6]. Importantly, MRD-guided therapy has already been established in patients with hematological malignancies and colorectal cancer [7]. In the context of UC, we extend the concept of DPO to encompass circulating tumor DNA (ctDNA)- and urinary tumor DNA (utDNA)-guided longitudinal monitoring at predefined clinical decision points, which has been validated in the first phase 3 study in this disease [8]. This approach relies on repeated real-time molecular assessments, primarily through liquid biopsy technologies, to longitudinally monitor tumor evolution, track clonal dynamics, and guide adaptive therapeutic strategies [9,10,11]. Structured around a “Concept–Evidence–Implementation” axis, this review introduces a novel clinical actionability matrix for dynamic decision making, synthesizes landmark clinical evidence, delineates enabling biomarker technologies, and explores analytical and implementation challenges of this emerging paradigm.

This non-systematic narrative review synthesized literature retrieved from PubMed/MEDLINE, Embase, Web of Science, and the Cochrane Library (January 2015–February 2026) databases, prioritizing landmark randomized controlled trials (RCTs), large prospective cohorts, and key translational studies. As a non-systematic approach, this methodology carries inherent selection bias, and some relevant studies may not have been captured.

## 2. Framework for Dynamic Decision Making

*Clinical Question: How can disease stage, temporal dynamics, and biomarkers be systematically integrated to inform actionable clinical decisions?*

To translate the concept of dynamic precision oncology into a practical clinical tool, we propose a unified clinical actionability framework that applies principles of ESMO Scale for Clinical Actionability of molecular Targets (ESMO-ESCAT) [12] and recognizes the clear gap between established evidence and hypothesis-generating concepts. The clinical utility of a biomarker depends fundamentally on the disease stage, histological subtype, molecular–genetic context (e.g., FGFR alterations, DDR pathway status), temporal context, therapeutic modality, and the specific clinical question [13]. Despite the growing adoption of liquid biopsy and molecular profiling technologies, clinicians currently lack systematic guidance on when and how to deploy specific biomarker tests. This gap leads to inconsistent test utilization, redundant testing, and missed opportunities to optimize therapy [14].

**Figure 1:**
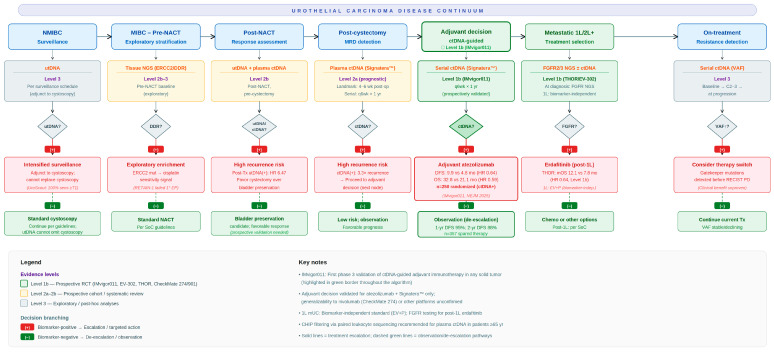
Circulating tumor DNA (ctDNA)-guided clinical decision-making algorithm across the urothelial carcinoma (UC) disease continuum. Seven sequential decision nodes span from non-muscle-invasive bladder cancer (NMIBC) surveillance to on-treatment resistance detection. Each node specifies the recommended testing modality (urinary tumor DNA (utDNA), plasma ctDNA, or tissue next-generation sequencing (NGS)), evidence Level, sampling timepoint, and biomarker-guided decision branch: positive results (red) direct treatment escalation; negative results (green) support de-escalation or observation. The adjuvant decision node (green border) is the only node supported by Level 1b evidence from the IMvigor011 trial, validated for atezolizumab with the Signatera™ assay (disease-free survival (DFS): hazard ratio (HR) 0.64; overall survival (OS): HR 0.59); generalizability to other agents or platforms is unconfirmed. First-line metastatic UC remains a biomarker-independent decision (enfortumab vedotin plus pembrolizumab (EV + P)). Fibroblast growth factor receptor (FGFR) 2/3 testing is required for post-first-line erdafitinib eligibility. All other nodes represent investigational applications (Level 2a–3) requiring prospective validation. Clonal hematopoiesis of indeterminate potential (CHIP) filtering via paired leukocyte sequencing is recommended for plasma ctDNA testing in patients aged ≥65 years. MRD, molecular residual disease; NACT, neoadjuvant chemotherapy; RECIST, Response Evaluation Criteria in Solid Tumors; SoC, standard of care; VAF, variant allele frequency.

We distinguish two levels of actionability relevant to clinical decision making. Table 1 presents the Current Evidence-Based Actionability Matrix, detailing biomarker-driven clinical decisions supported by Level 1–2a evidence, which has been integrated into clinical practice. Table 2 presents the Exploratory and Future Actionability Matrix, which outlines key research questions and dynamic concepts, such as minimal residual disease (MRD)-guided therapy de-escalation, currently supported by Level 3 or exploratory evidence requiring prospective validation before clinical implementation [7,15]. This structured approach fundamentally shifts the paradigm from simply “ordering a ctDNA test” to the more nuanced approach of “utilizing a specific biomarker at a specific time to answer a specific question,” thereby providing more granular and actionable results for guiding clinical practice. The integration of these decision points across the disease continuum is illustrated in Figure 1.

**Table 1:** Current Evidence-Based Actionability Matrix.

Actionability Category	Stage/Setting	Clinical Decision Point	Biomarker	Biomarker Specification/Assay Type	Recommended Action	Level of Evidence	Key Findings	Limits/Notes
Biomarker-independent	mUC–1L (all newcomers), EV + P	Initial Treatment	N/A (subgroup benefit across PD-L1, Nectin-4)	N/A	Initiate EV + P for most patients	1b	EV-302: mOS 33.8 vs. 15.9 months; HR 0.51 [16].	Benefit seen in all-comers, diminishing need for 1L predictive biomarker
Biomarker-independent	mUC–1L (cisplatin-eligible), Nivo + GC	Initial Treatment (if EV + P contraindicated)	N/A	N/A	Nivolumab + GC	1b	CheckMate 901: OS HR 0.78 [17].	An option, but the magnitude of OS benefit is markedly less than EV + P
Biomarker-selected	mUC–post-1L, *FGFR2/3* altered	*FGFR2/3* altered	*FGFR2/3* (tissue/ctDNA)	NGS (tissue or ctDNA-based)	Erdafitinib	1b	THOR: mOS 12.1 vs. 7.8 months; HR 0.64 [18].	THOR trial (phase 3) confirmed OS benefit vs. chemotherapy
	Localized MIBC–adjuvant (unselected)	Adjuvant therapy	N/A (benefit also in PD-L1 ≥1%)	N/A	Adjuvant nivolumab	Prognostic: 2a; Predictive: 1b (IMvigor011)	CheckMate 274 positive for DFS; durable benefit on extended follow-up [19].	Clinical Paradox: Regulatory Approval: supports treating all high-risk patients (unselected). Exploratory Data (e.g., IMvigor010): Suggests benefit is concentrated in the ctDNA(+) subset. Key Issue: Raises questions about potential overtreatment in ctDNA(−) patients.
Biomarker-stratified (exploratory)	Localized MIBC–post-op prognostication	Prognostication (risk stratification)	ctDNA (landmark MRD)	Tumor-informed (personalized) assay	If (+) = high risk; if (−) = low risk	2a (prognostic utility established); 3 (predictive utility investigational)	Multiple prospective cohorts/meta-analyses show strong prognostic value; predictive utility now by exploratory post hoc analyses from IMvigor010 [11].	Prognostic value: Strong; established in multiple prospective cohorts (Level 2a). Predictive value: IMvigor010 (Exploratory, post hoc, Level 3): Treatment benefit observed only in ctDNA(+) subset (HR 0.59). Suggests biological plausibility for MRD-guided therapy. Requires prospective validation (IMvigor011, ongoing). Current Status: Validated prognostic biomarker (Level 2a). Predictive utility now confirmed by IMvigor011 (see below), but specific to serial Signatera™ monitoring and atezolizumab only.
Biomarker-stratified	Localized MIBC–post-cystectomy (ctDNA-guided)	MRD-guided adjuvant therapy selection	ctDNA (serial MRD)	Tumor-informed (personalized) assay; Signatera™ (16-variant multiplex PCR); serial testing q6wk ×1yr	If ctDNA(+): adjuvant atezolizumab. If ctDNA(−): observation (treatment de-escalation)	1b	IMvigor011: ctDNA(+) patients (n = 250) randomized to atezolizumab vs. placebo. DFS: 9.9 vs. 4.8 months (HR 0.64, *p* = 0.0047). OS: 32.8 vs. 21.1 months (HR 0.59, *p* = 0.0131). ctDNA(−) patients (n = 357) spared therapy: 1-yr DFS 95%, 2-yr DFS 88% [8].	First phase 3, Level 1 evidence for ctDNA-guided adjuvant immunotherapy in any solid tumor. Validates both treatment escalation (ctDNA+) and de-escalation (ctDNA−). Limitations: Single assay platform (Signatera™); generalizability to other ctDNA assays unvalidated. Fixed q6wk sampling protocol; optimal frequency in routine practice undefined. Atezolizumab-specific; applicability to nivolumab (CheckMate 274 setting) requires separate validation.

Abbreviations: ctDNA, circulating tumor DNA; DFS, disease-free survival; EV + P, enfortumab vedotin + pembrolizumab; *FGFR2/3*, fibroblast growth factor receptor 2/3; GC, gemcitabine-cisplatin; HR, hazard ratio; MIBC, muscle-invasive bladder cancer; MRD, molecular residual disease; Nivo, nivolumab; mOS, median overall survival; mUC, metastatic urothelial carcinoma; NGS, next-generation sequencing; OS, overall survival; PD-L1, programmed death-ligand 1; 1L, first-line. Entries marked “N/A (biomarker)” delineate settings where biomarker-independent treatment is the current standard, contextualizing when dynamic molecular guidance is and is not required.

**Table 2:** Applications of Investigational Biomarkers that Require Prospective Validation.

Actionability Category	Stage/Setting	Clinical Decision Point	Biomarker	Proposed Action	Level of Evidence	Limits/Notes
Biomarker-stratified (investigational)	NMIBC–Surveillance	Recurrence Monitoring	utDNA	Adjunct to cystoscopy	3	Assays heterogeneous; optimal intervals undefined. UroScout: 100% sensitivity ≥T1 [20]. Cannot yet safely replace cystoscopy [21].
Biomarker-stratified (investigational)	NMIBC/MIBC–Diagnosis	Non-invasive detection and risk stratification	Urine cfDNA	Pre-screen before invasive workup	2b	Sensitivity 86.7%, specificity 99.3%, CHIP-free [22]. CLIA/CE-marked platforms emerging [23,24]. Clinical impact unvalidated.
Biomarker-stratified (investigational)	MIBC–Pre-NACT	Predict NACT response	ERCC2 (DDR)	Exploratory enrichment marker only	2b-3	Retrospective signal [25]. RETAIN-1 failed primary endpoint → biomarker-guided cystectomy omission not supported [26].
Biomarker-stratified (investigational)	MIBC–Pre-/Post-NACT	Guide bladder preservation	ctDNA + utDNA (dual)	Combined local (urine) + systemic (plasma) monitoring	2b	utDNA detection 89% vs. plasma 43% [27]. Post-treatment utDNA(+): HR 6.47 for recurrence [28]. No confirmatory RCTs [27].
Biomarker-stratified (investigational)	MIBC–Post-operative	MRD-guided adjuvant (non-validated combinations)	ctDNA (landmark)	ctDNA(+): adjuvant ICI; ctDNA(−): observation	1b atezo/Signatera™ (Table 1); 3 for other agents/platforms	IMvigor011 validated one combination only. Nivolumab and alternative assays require independent validation.
Biomarker-stratified (investigational)	MIBC–Surveillance	Molecular Relapse	Longitudinal ctDNA	ctDNA conversion → early intervention within trial	2a (prognosis)	Lead time up to 131 days over imaging [29]. Optimal intervention strategy undefined [30].
Biomarker-stratified (investigational)	mUC–On-treatment	Resistance detection	ctDNA (serial VAF)	Monitor resistance mutations → consider therapy switch	3	Detects FGFR gatekeeper mutations before RECIST progression [31]. Whether early switch improves outcomes is unknown.

All entries require prospective validation before clinical implementation outside research protocols. Abbreviations: cfDNA, cell-free DNA; CHIP, clonal hematopoiesis of indeterminate potential; ctDNA, circulating tumor DNA; DDR, DNA damage repair; ERCC2, excision repair cross-complementation group 2; HR, hazard ratio; ICI, immune checkpoint inhibitor; MIBC, muscle-invasive bladder cancer; MRD, molecular residual disease; NACT, neoadjuvant chemotherapy; NMIBC, non-muscle-invasive bladder cancer; RCT, randomized controlled trial; utDNA, urinary tumor DNA; VAF, variant allele frequency.

## 3. Landmark Therapeutic Advances Are Reshaping Clinical Practice

*Clinical Question: What is the highest-Level evidence guiding therapy across the disease continuum?*

### 3.1. First-Line Metastatic Urothelial Carcinoma

Two pivotal trials have redefined the first-line treatment landscape. The EV-302/KEYNOTE-A39 trial established enfortumab vedotin plus pembrolizumab as the new global standard of care, with updated median overall survival (OS) of 33.8 versus 15.9 months for chemotherapy (hazard ratio [HR] 0.51, 95% confidence interval [CI] 0.43–0.61) [16,32]. Importantly, this benefit was consistently observed across all predefined subgroups, including those ineligible for cisplatin, patients with varying levels of PD-L1 expression, and those with poor-prognosis visceral metastases [33]. The CheckMate 901 trial demonstrated a more modest benefit for nivolumab plus gemcitabine-cisplatin in cisplatin-eligible patients (median OS 21.7 vs. 18.9 months; HR 0.78, 95% CI 0.63–0.96) [17]. Notably, the magnitude of EV-302 survival benefit renders predictive biomarker selection unnecessary in this setting, representing a biomarker-independent standard. The CheckMate 901 trial remains a reasonable alternative when enfortumab vedotin-based therapy is contraindicated or unavailable [17,34].

Thus, first-line metastatic UC is currently a biomarker-independent treatment decision, and dynamic molecular guidance is not required for initial therapy selection [34].

### 3.2. Adjuvant Setting and the MRD Evidence Conundrum

The adjuvant setting exemplifies the evolution from unselected to biomarker-guided therapy and represents the most compelling application of dynamic precision oncology in patients with UC.

The CheckMate 274 trial established adjuvant treatment with nivolumab for all patients considered high-risk MIBC after radical cystectomy, demonstrating durable disease-free survival (DFS) benefit in both the intent-to-treat population (HR 0.71, 95% CI 0.58–0.86) and in the PD-L1 ≥1% subgroup (HR 0.52, 95% CI 0.37–0.72) at a median follow-up of 36.1 months [19]. This led to regulatory approval supporting treatment of all eligible patients regardless of biomarker status. However, the exploratory ctDNA analysis from the CheckMate 274 trial revealed that ctDNA(+) patients had a 3.3-fold higher risk of recurrence, raising questions about potential overtreatment in the ctDNA(−) population [15].

The IMvigor010 adjuvant atezolizumab trial failed in the unselected intent-to-treat population (HR 0.89, 95% CI 0.74–1.08) [35]. However, a post hoc biomarker analysis revealed a striking treatment–biomarker interaction: ctDNA(+) patients derived significant OS benefit from atezolizumab (HR 0.59, 95% CI 0.41–0.86), whereas ctDNA(−) patients showed no benefit [11,36]. This generated the hypothesis that MRD status could guide adjuvant therapy selection, a conclusion that is biologically compelling but requires prospective validation.

The IMvigor011 trial resolved this conundrum by providing the first Level 1b evidence for adjuvant immunotherapy guided by ctDNA in any solid tumor. In this phase 3 randomized double-blind trial, 761 patients with post-cystectomy MIBC without radiographic evidence of residual disease underwent serial ctDNA monitoring using the Signatera™ tumor-informed assay every 6 weeks for up to 1 year. Of these, 250 patients who tested ctDNA(+) were randomized 2:1 to atezolizumab versus placebo, whereas 357 patients who remained persistently ctDNA(−) received no adjuvant therapy [8].

The results were practice-changing. Atezolizumab significantly improved DFS compared with placebo in patients with ctDNA(+) (median 9.9 vs. 4.8 months; HR 0.64, 95% CI 0.47–0.87; *p* = 0.0047) and, critically, also improved OS (median 32.8 vs. 21.1 months; HR 0.59, 95% CI 0.39–0.90; *p* = 0.0131). Meanwhile, patients who were ctDNA(−) and were spared adjuvant therapy achieved 1-year DFS of 95% and 2-year DFS of 88%, with over 90% alive at the median follow-up, confirming the strong negative predictive value of serial ctDNA monitoring and validating treatment de-escalation [8].

IMvigor011 transitioned ctDNA from a prognostic biomarker to a validated predictive tool for the selection of adjuvant treatments in patients with MIBC. However, important limitations must be recognized. The validation is assay-specific (Signatera™) and agent-specific (atezolizumab); generalizability to nivolumab (the CheckMate 274 agent) or alternative ctDNA platforms remains unconfirmed [10,15]. The fixed 6-week serial monitoring schedule represents the only prospectively validated sampling strategy. The ongoing IMvigor011 companion study and future platform-comparison trials will be critical to define the broader applicability of this paradigm. The relationship between these pivotal adjuvant trials is summarized in Figure S1, and the corresponding evidence hierarchy is detailed in Table 3.

IMvigor011 thus establishes ctDNA-guided MRD status as the first phase 3–validated predictive biomarker framework for adjuvant therapy selection in a solid tumor [8].

### 3.3. Molecularly Selected Refractory Disease

In patients experiencing metastatic UC progressing after platinum chemotherapy and immunotherapy, molecular selection is paramount. The THOR trial provided definitive Level 1 evidence for targeted therapy in genomically defined subsets: patients with susceptible FGFR2 or FGFR3 alterations randomized to erdafitinib versus chemotherapy demonstrated a clinically meaningful OS benefit (median 12.1 vs. 7.8 months; HR 0.64, 95% CI 0.47–0.88; *p* = 0.005), with an objective response rate of 45.6% versus 11.5% [18]. These results confirmed erdafitinib as the standard of care in this population and established the imperative for routine FGFR testing in all patients with metastatic UC [37].

Beyond initial molecular selection, ctDNA enables the detection of secondary resistance mechanisms. Exploratory analyses in erdafitinib-treated patients have shown that ctDNA can identify the emergence of FGFR gatekeeper mutations weeks to months before radiographic progression [38]. However, this remains a technically feasible observation rather than a clinically validated strategy: whether ctDNA-triggered therapy switching improves outcomes compared with switching at standard RECIST progression is currently unknown and requires prospective investigation.

Additionally, HER2 (ERBB2) expression assessed by immunohistochemistry identifies patients eligible for trastuzumab deruxtecan, which received FDA pan-tumor accelerated approval for IHC 3+ unresectable or metastatic solid tumors including UC (Level 2b evidence) [39,40]. These actionable biomarker–therapy pairs are detailed in Table 5.

The refractory setting demonstrates the value of tissue-based genomic selection (FGFR testing) and emerging ctDNA-based resistance monitoring, although only the former is currently validated for clinical decision making.

**Table 3:** Evidence Hierarchy of Key Studies in Dynamic Precision Oncology for Urothelial Carcinoma.

Trial	Design	N	Evidence	Key Findings
EV-302/KEYNOTE-A39	phase 3 RCT	886	1b	EV + P vs. chemotherapy: mOS 33.8 vs. 15.9 mo; HR 0.51. New 1L standard. Biomarker-independent [16].
CheckMate 901	phase 3 RCT	608	1b	Nivo + GC vs. GC: mOS 21.7 vs. 18.9 mo; HR 0.78. Cisplatin-eligible only. Biomarker-independent [17].
THOR	phase 3 RCT	266	1b	Erdafitinib vs. chemotherapy (FGFR2/3-altered): mOS 12.1 vs. 7.8 mo; HR 0.64. Biomarker-selected [18].
CheckMate 274	phase 3 RCT	709	1b	Adj nivo vs. placebo: DFS HR 0.71 (ITT), 0.52 (PD-L1 ≥ 1%). Biomarker-independent approval [19].
IMvigor011 ^1^	phase 3 RCT (ctDNA-guided)	250 randomized/761 screened	1b	ctDNA(+): atezo vs. placebo—DFS HR 0.64 (*p* = 0.0047); OS HR 0.59 (*p* = 0.0131). ctDNA(−): 1-yr DFS 95%. First Level 1 ctDNA-guided adjuvant validation [8].
CheckMate 274 (ctDNA) ^2^	Exploratory from phase 3	353	Prognostic 2a; Predictive 3 → 1b *	ctDNA(+): 3.3× risk of recurrence. Predictive hypothesis now validated by IMvigor011 * [15].
IMvigor010 substudy ^2^	Prospective + biomarker	581	Prognostic 2a; Predictive 3 → 1b *	ctDNA(+): DFS HR 6.3 (prognostic); treatment HR 0.59 (exploratory). Validated by IMvigor011 * [36].
Gao et al. ^3^	Meta-analysis	1088	2a	ctDNA prognostic: pooled OS HR 4.51; *I*^2^ = 80%. Cross-platform extrapolation limited [41].
Nordentoft et al.	Prospective cohort	112	2b	WGS-based AI platform (C2i): 91% sensitivity, 92% specificity for MRD; 131-day lead over imaging [29].

Abbreviations: AI, artificial intelligence; Adj, adjuvant; ctDNA, circulating tumor DNA; DFS, disease-free survival; EV + P, enfortumab vedotin + pembrolizumab; FGFR2/3, fibroblast growth factor receptor 2/3; GC, gemcitabine-cisplatin; HR, hazard ratio; ITT, intention-to-treat; mOS, median overall survival; Nivo, nivolumab; OS, overall survival; PD-L1, programmed death-ligand 1; RCT, randomized controlled trial; WGS, whole-genome sequencing; 1L, first-line. * Predictive hypothesis validated by IMvigor011 for atezolizumab/Signatera™ specifically; cross-agent and cross-platform generalizability unconfirmed. Level: Definition; 1b: Individual RCT; 2a: Systematic review of cohort studies or high-quality prospective cohorts; 2b: Individual cohort study or low-quality RCT; 3: Case-control or exploratory/post hoc analyses; Prognostic = risk stratification regardless of treatment. Predictive = identifies treatment-specific benefit (requires prospective validation). ^1^. IMvigor011: Assay-specific (Signatera™) and agent-specific (atezolizumab). Serial q6wk monitoring protocol. Applicability to nivolumab or other ctDNA platforms requires separate validation. ^2^. CheckMate 274 ctDNA/IMvigor010 substudy: originally exploratory (Level 3). Predictive utility now prospectively confirmed by IMvigor011, but only for atezolizumab + Signatera™ combination. ^3^. Gao et al.: *I*^2^ = 80% reflects heterogeneous assay types and sampling timepoints. Pooled metrics should not be extrapolated across platforms.

## 4. Enabling Technologies for Real-Time Molecular Interrogation

*Clinical Question: Which tools enable dynamic monitoring, and what are the requirements for clinical implementation?*

### 4.1. Circulating Tumor DNA: Principles and Performance

The realization of dynamic precision oncology depends on technologies enabling safe, frequent, and noninvasive tumor molecular interrogation [42]. Circulating tumor DNA assays fall into two categories with distinct trade-offs [43]. Tumor-informed assays begin with comprehensive tumor tissue sequencing to identify 16–50 clonal mutations, then a personalized multiplex PCR assay is designed to track these variants in serial plasma samples. This approach achieves superior analytical sensitivity (limit of detection ~0.01% VAF), making it the current gold standard for MRD detection and the platform validated in the IMvigor011 trial [44]. However, this approach requires upfront tissue and a longer initial turnaround time (3–5 weeks). In contrast, tumor-agnostic assays employ fixed next-generation sequencing panels to screen hundreds of cancer-associated mutations directly from plasma without prior tumor sequencing. Although faster (7–10 days) and logistically simpler, this approach is generally less sensitive for MRD detection (~0.1% VAF) [45]. The practical trade-offs between representative platforms are summarized in Table 4.

A critical challenge undermining ctDNA specificity is clonal hematopoiesis of indeterminate potential (CHIP). CHIP-derived mutations in genes such as DNMT3A, TET2, and TP53 create false-positive results [46]. This is particularly concerning in UC given a median diagnosis age of 73 years, with CHIP affecting 10–15% of individuals over 65 and up to 20% over 80 years [47]. Correction requires concurrent paired leukocyte DNA sequencing, adding approximately USD 500–1000 per sample [48]. For UC patients over 65 years, CHIP filtering should be considered mandatory for any ctDNA testing guiding treatment decisions [49].

**Table 4:** Comparison of Representative ctDNA Platforms for MRD Detection.

Feature	Signatera™ (Natera)	Guardant Reveal™ (Guardant Health)
Methodology	Tumor-Informed (Personalized) Requires WES of tumor + matched normal Creates custom multiplex PCR assay (16 variants)	Tumor-Agnostic (Tissue-Free) Fixed panel (no tumor tissue required) Uses genomic alterations + methylation signature
Analytical Sensitivity (LOD)	Very High: ~0.01% VAF	High: ~0.1% VAF LOD may vary based on analyte (genomic vs. methylation)
Typical Turnaround Time (TAT)	Slow (3–5 weeks) 2–3 weeks for initial tumor sequencing/assay design 1–2 weeks for plasma sample analysis	Fast (approx. 7–10 days) No upfront tissue analysis needed
Key Clinical Validation in UC	Strong (Level 2a/3) Used in IMvigor010 (post hoc) Used in IMvigor011 (prospective) Multiple prospective cohorts	Emerging Primary validation in colorectal, breast, and lung cancer
Key Trade-Off	Pro: Highest sensitivity; strong UC validation. Con: Requires tissue; long initial TAT; higher cost.	Pro: Fast TAT; no tissue needed (logistically simpler). Con: Lower sensitivity; less UC-specific validation.

Abbreviations: ctDNA, circulating tumor DNA; LOD, limit of detection; MRD, molecular residual disease; PCR, polymerase chain reaction; TAT, turnaround time; UC, urothelial carcinoma; VAF, variant allele frequency; WES, whole exome sequencing.

### 4.2. Urine-Based Liquid Biopsy: A Complementary Modality for Urothelial Carcinoma

Given the unique anatomical context of UC, urine represents a disease-proximal biofluid with inherent advantages over plasma. Tumor cells shed DNA directly into the urinary tract, yielding higher tumor DNA concentrations locally while avoiding the dilution and CHIP confounding that limit plasma-based approaches [22]. In patients with newly diagnosed UC, urinary tumor DNA (utDNA) achieved sensitivity of 86.7% and specificity of 99.3% without CHIP interference, whereas concurrent plasma ctDNA analysis was confounded by hematopoietic variants [22]. In the MIBC neoadjuvant setting, utDNA was detected in 89% of pre-treatment samples compared with only 43% for plasma ctDNA, demonstrating approximately twofold higher detection rates [27].

Several platforms are advancing toward clinical implementation. UroAmp™ (CLIA-certified) reported 95% sensitivity with 100% for high-grade and muscle-invasive disease across a multicenter validation of 581 patients [24]. The Inform-Bladder test (CE-marked) achieved 97.4% sensitivity for grade 3 tumors in the BladderPath study of 884 samples [23]. UroScout, utilizing high-volume (100 mL) urine collection, reported 100% sensitivity for ≥T1 and carcinoma in situ with feasible home mail-in collection [20].

For bladder-sparing approaches, combined urine and plasma monitoring provides complementary information: utDNA reflects residual intravesical disease, whereas plasma ctDNA captures systemic dissemination. In clinical complete responders, detectable utDNA predicted shorter bladder-intact survival (HR 6.47; *p* = 0.008) [28]. This dual-analyte strategy may represent the optimal liquid biopsy approach for UC and is unique among solid tumors [27,28]. Standardization challenges remain, including the optimal urine collection volume, processing protocols, and the absence of prospective utility trials demonstrating that urine-based monitoring can safely modify cystoscopy schedules or inform treatment decisions [50].

### 4.3. Actionable Biomarker-Therapy Pairs in Current Practice

Three actionable biomarker-therapy pairs are currently relevant to management of patients with UC. Nectin-4 is expressed in over 90% of UC tumors, rendering enfortumab vedotin a biomarker-independent therapy requiring no companion diagnostic testing; Level 1 evidence from EV-302 supports its use with pembrolizumab in first-line metastatic UC [32].

FGFR2/3 alterations, detected by tissue-based RT-PCR or NGS, identify patients eligible for erdafitinib; susceptible alterations include FGFR3 mutations (e.g., S249C, Y373C) and FGFR2/3 fusions, with Level 1 evidence from the THOR trial supporting erdafitinib following progression on platinum-based chemotherapy and checkpoint inhibitor therapy [18,51].

HER2 (ERBB2) expression assessed by immunohistochemistry identifies patients eligible for trastuzumab deruxtecan, which received FDA pan-tumor accelerated approval for IHC 3+ unresectable or metastatic solid tumors including UC (Level 2b evidence), with clinical activity also observed in IHC 2+ tumors [40]. These biomarker-therapy pairs, including testing methods and key toxicities, are detailed in Table 5.

### 4.4. Artificial Intelligence in Dynamic Molecular Monitoring

Artificial intelligence is increasingly integrated into liquid biopsy analysis, addressing key analytical challenges across three domains.

#### 4.4.1. AI-Enhanced ctDNA Detection

Machine-learning algorithms substantially improve signal-to-noise ratios in ctDNA analysis. MRD-EDGE, a deep learning framework, achieves approximately 300-fold signal enrichment for single nucleotide variant (SNV) detection, enabling plasma-only MRD monitoring without prior tumor tissue sequencing [52]. Fragle, a deep learning model utilizing DNA fragment length distributions from low-pass whole-genome sequencing, provides tumor-agnostic ctDNA quantification that eliminates the need for matched tissue entirely [53]. These tumor-agnostic AI approaches have the potential to reduce costs and logistical barriers associated with tumor-informed assays.

#### 4.4.2. Fragmentomics-Based Monitoring

The DELFI-TF classifier estimates tumor fraction from cell-free DNA fragmentation patterns without requiring mutation identification, serving as an independent predictor of OS (HR 9.84; *p* < 0.0001) [54]. Similarly, multi-cancer early detection platforms integrating artificial intelligence (AI) with cfDNA fragmentomics have demonstrated bladder cancer detection with an AUC of 0.96 in a multicenter validation study [55].

#### 4.4.3. UC-Specific Applications

In bladder cancer, whole-genome-sequencing-based computational platforms have demonstrated direct clinical relevance. The C2i Genomics platform achieved 91% sensitivity and 92% specificity for post-cystectomy MRD detection in 112 patients with MIBC, with a median lead time of 131 days over conventional imaging [29]. For treatment prediction, multimodal deep learning integrating histopathology with gene expression data achieved an AUC of approximately 0.80 for predicting neoadjuvant chemotherapy response in MIBC, substantially outperforming unimodal approaches [56]. Despite these advances, prospective clinical validation, regulatory approval pathways, and algorithmic interpretability remain prerequisites before clinical adoption [56].

### 4.5. Standardization Roadmap and Clinical Trial Design Framework

*Clinical Question: What standardization requirements must be met to translate dynamic monitoring into routine clinical practice?*

#### 4.5.1. Analytical Standardization

The Association for Molecular Pathology and College of American Pathologists (AMP/CAP) joint consensus provides 13 recommendations for cell-free DNA assay validation, covering pre-analytical variables, limit of detection, analytical sensitivity and specificity, CHIP interference assessment, and reporting standards [57]. BloodPAC has published the first generic analytical validation protocols specifically for tumor-informed MRD assays, defining requirements for limit of blank, limit of detection, accuracy, and precision, developed with FDA CDRH presubmission feedback [58]. The International Society of Liquid Biopsy (ISLB) has established minimum quality requirements for institutions performing ctDNA testing, covering pre-analytical through post-analytical standardization [59].

#### 4.5.2. Clinical Response Criteria

Liquid Biopsy RECIST (LB-RECIST) guidelines provide standardized molecular response definitions: ctDNA Complete Response, Partial Response, Stable Disease, and Progressive Disease, using aggregate variant allele frequency as the primary quantitative metric [60]. These criteria have been clinically validated in metastatic colorectal cancer and are being extended to other tumor types [61].

#### 4.5.3. Regulatory Framework

The November 2024 FDA guidance on ctDNA use in early-stage solid tumor drug development addresses ctDNA for patient enrichment, treatment response monitoring, and as an early endpoint potentially supporting drug approval, including via the accelerated approval pathway [62].

#### 4.5.4. Proposed Trial Design for UC

Based on these frameworks and the IMvigor011 prototype, we propose that future biomarker-guided trials involving patients with UC incorporate the following elements: (i) prospective randomized biomarker-strategy design with ctDNA-guided arm versus standard of care; (ii) analytically validated tumor-informed assays with LOD ≤ 0.01% VAF; (iii) fixed serial sampling at 6-week intervals as per the IMvigor011 protocol; (iv) mandatory CHIP filtering via paired leukocyte sequencing for patients aged ≥65 years; (v) hierarchical DFS/OS endpoints with molecular response per LB-RECIST as secondary endpoint; (vi) concurrent ctDNA(−) observation arm to validate de-escalation safety; and (vii) consideration of dual-analyte monitoring (urine + plasma) for intravesical disease settings [8,57,58,59,60]. These elements are summarized in Table 6.

**Table 5:** Actionable Biomarker-Therapy Pairs in Urothelial Carcinoma.

Target/Biomarker	Approved Agent(s)	Test Method(s)	Positivity Criteria	Level of Evidence	Clinical Setting/Line of Therapy	Key Toxicities to Monitor	Reference
Nectin-4	Enfortumab vedotin	Not required for treatment eligibility.	N/A; considered an “all-comers” target due to expression in >90% of UC.	Level 1b (phase 3 RCT showing OS/PFS benefit in all-comers)	1L mUC (with Pembrolizumab); 2L+ mUC (monotherapy).	Peripheral neuropathy, skin rash, hyperglycemia, ocular disorders.	[16,32,63]
*FGFR2/3* Alterations	Erdafitinib	Tissue-based RT-PCR (e.g., Therascreen^®^ FGFR Kit) or DNA/RNA-based NGS.	Susceptible *FGFR3* mutations (e.g., S249C, Y373C) or *FGFR2/3* fusions (e.g., *FGFR3*-TACC3).	Level 1b (phase 3 RCT vs. chemotherapy with OS benefit)	mUC post-platinum and post-ICI therapy.	Hyperphosphatemia, central serous retinopathy, stomatitis, nail/skin disorders.	[18]
*HER2* (*ERBB2*) Expression	Trastuzumab Deruxtecan (T-DXd)	Immunohistochemistry (IHC) on tumor tissue.	U.S. FDA pan-tumor approval for IHC 3+. Clinical activity also seen in IHC 2+.	Level 2b (prospective multi-cohort/single-arm evidence; tumor-agnostic accelerated approval)	Unresectable or metastatic solid tumors (including UC) post-prior therapy.	Interstitial lung disease/pneumonitis (Boxed Warning), nausea, myelosuppression, fatigue.	[39,40]

Abbreviations: *ERBB2*, erb-b2 receptor tyrosine kinase 2; FDA, Food and Drug Administration; *FGFR2/3*, fibroblast growth factor receptor 2/3; *HER2*, human epidermal growth factor receptor 2; ICI, immune checkpoint inhibitor; IHC, immunohistochemistry; mUC, metastatic urothelial carcinoma; NGS, next-generation sequencing; OS, overall survival; PFS, progression-free survival; RCT, randomized controlled trial; RT-PCR, reverse transcription polymerase chain reaction; T-DXd, trastuzumab deruxtecan; UC, urothelial carcinoma; 1L, first-line; 2L+, second-line and beyond.

**Table 6:** Proposed Framework for ctDNA-Guided Clinical Trials in UC.

**Panel A:** Core Trial Design Elements			
**Domain**	**Element**	**Recommendation**	**Validated Exemplar/Reference**
**Study Design**	Overall architecture	Prospective, randomized, biomarker-strategy design: ctDNA-guided arm vs. standard-of-care	IMvigor011
	Randomization	ctDNA(+): randomize to intervention vs. control. ctDNA(−): observation arm with protocol-defined safety monitoring	IMvigor011
	Blinding	Double-blind preferred for adjuvant setting; open-label acceptable for metastatic adaptive designs	IMvigor011
**Assay Requirements**	Platform type	Tumor-informed (personalized) assay preferred for MRD settings	Signatera™/IMvigor011
	Analytical sensitivity	Limit of detection (LOD) ≤0.01% variant allele frequency (VAF) for MRD; ≤0.1% VAF acceptable for metastatic monitoring	BloodPAC
	Analytical validation	Limit of blank (LOB), LOD, accuracy, precision, and reproducibility should follow generic MRD assay validation protocols	BloodPAC
	CHIP filtering	Paired leukocyte sequencing recommended; particularly important in older patients	AMP/CAP
	Institutional requirements	Minimum institutional quality standards and participation in external quality assessment programs recommended	ISLB
**Sampling Protocol**	Baseline	Pre-treatment (pre-surgery or pre-neoadjuvant chemotherapy (NACT))	—
	Landmark MRD	4–6 weeks post-cystectomy	IMvigor011
	Serial monitoring	Every 6 weeks for year 1	IMvigor011
	Extended surveillance	Every 3 months years 1–2; every 6 months years 3–5	Proposed
	On-treatment (metastatic)	Baseline → cycle 2–3 (early response) → at progression	Proposed
**Endpoints**	Primary	Disease-free survival (DFS) for adjuvant trials; progression-free survival (PFS) or overall survival (OS) for metastatic trials	FDA guidance
	Key secondary	OS (adjuvant); molecular response per liquid biopsy RECIST (LB-RECIST) in both settings	LB-RECIST
	Exploratory	Molecular DFS (time to ctDNA conversion); ctDNA clearance rate; lead time over imaging	Proposed
**Dual-Analyte Option**	Urine + plasma	Consider concurrent urinary tumor DNA (utDNA) for trials involving intravesical disease, including non-muscle-invasive bladder cancer (NMIBC) surveillance and bladder-preservation settings	Christensen et al.; Galsky et al.
**Panel B:** Molecular Response Criteria (per LB-RECIST)			
**Category**	**Abbreviation**	**Definition**	**Clinical Interpretation**
ctDNA Complete Response	CCR	Aggregate variant allele frequency (aggVAF) becomes undetectable from a detectable baseline	Favorable; associated with improved outcomes
ctDNA Partial Response	CPR	≥50% decrease in aggVAF from baseline	Intermediate favorable
ctDNA Stable Disease	CSD	Neither CPR nor ctDNA Progressive Disease (CPD) criteria are met	Requires continued monitoring
ctDNA Progressive Disease	CPD	≥50% increase in aggVAF from nadir, or conversion from undetectable to detectable ctDNA	Unfavorable; consider treatment reassessment
**Panel C:** Pre-analytical Specifications for ctDNA Testing			
**Step**	**Specifications**		**Failure Impact**
Blood collection tube	Cell-free DNA stabilizing tube preferred when immediate processing is not feasible		Genomic DNA contamination, leading to false-positive results
Transport time	Follow manufacturer-validated stability windows for stabilizing tubes; rapid processing required for ethylenediaminetetraacetic acid (EDTA) tubes		cfDNA degradation or leukocyte lysis
Plasma separation	Double centrifugation recommended		Cellular DNA contamination
cfDNA input	Assay-specific minimum input should be predefined and analytically validated		Reduced sensitivity below LOD threshold
Urine collection (if dual-analyte)	Standardized first-morning void or high-volume collection with preservative		utDNA degradation and reduced yield
Storage	Plasma and extracted cfDNA should follow validated assay-specific storage conditions		Analyte degradation over time

Abbreviations: aggVAF, aggregate variant allele frequency; AMP/CAP, Association for Molecular Pathology/College of American Pathologists; CCR, ctDNA complete response; cfDNA, cell-free DNA; CHIP, clonal hematopoiesis of indeterminate potential; CPD, ctDNA progressive disease; CPR, ctDNA partial response; CSD, ctDNA stable disease; ctDNA, circulating tumor DNA; DFS, disease-free survival; EDTA, ethylenediaminetetraacetic acid; FDA, Food and Drug Administration; ISLB, International Society of Liquid Biopsy; LB-RECIST, Liquid Biopsy Response Evaluation Criteria in Solid Tumors; LOB, limit of blank; LOD, limit of detection; MRD, molecular residual disease; NACT, neoadjuvant chemotherapy; NMIBC, non-muscle-invasive bladder cancer; OS, overall survival; PFS, progression-free survival; UC, urothelial carcinoma; utDNA, urinary tumor DNA; VAF, variant allele frequency. **Table footnote:** This framework integrates the validated ctDNA-guided design of IMvigor011 with current analytical, regulatory, and molecular-response frameworks from BloodPAC, AMP/CAP, ISLB, LB-RECIST, FDA ctDNA guidance, and dual-analyte urine/plasma studies in urothelial carcinoma [8,28,57,58,59,60,62].

## 5. Conclusions

The validation of ctDNA-guided adjuvant immunotherapy through the IMvigor011 trial marks a milestone for dynamic precision oncology in MIBC, demonstrating improvements in both DFS and OS. This milestone transitions dynamic precision oncology from concept to evidence-based practice. However, clinical implementation requires adherence to established standardization frameworks: analytically validated assays per BloodPAC and AMP/CAP guidelines, fixed sampling protocols, mandatory CHIP filtering, and molecular response assessment per LB-RECIST criteria. The integration of urine-based liquid biopsy offers a complementary CHIP-free modality uniquely suited to UC, whereas AI-driven analytical tools hold promise to enhance detection sensitivity and enable tumor-agnostic monitoring approaches. Future biomarker-guided trials should adopt prospective randomized designs with hierarchical endpoints, as exemplified by the IMvigor011 prototype. Economic and access barriers warrant dedicated investigation to ensure equitable implementation.

## Data Availability

No new data were created or analyzed in this study. Data sharing is not applicable to this article.

## Supplementary Materials

The following supporting information can be downloaded at: https://www.mdpi.com/article/10.3390/ijms27083474/s1.

## Abbreviations

The following abbreviations are used in this manuscript:

1L	first-line
2L+	second-line and beyond
aggVAF	aggregate variant allele frequency
AI	artificial intelligence
AMP/CAP	Association for Molecular Pathology/College of American Pathologists
AUC	area under the curve
AUROC	area under the receiver operating characteristic curve
CCR	ctDNA complete response
CE	Conformité Européenne
cfDNA	cell-free DNA
CHIP	clonal hematopoiesis of indeterminate potential
CI	confidence interval
CLIA	Clinical Laboratory Improvement Amendments
CPD	ctDNA progressive disease
CPR	ctDNA partial response
CSD	ctDNA stable disease
ctDNA	circulating tumor DNA
DDR	DNA damage repair
DFS	disease-free survival
DPO	dynamic precision oncology
EDTA	ethylenediaminetetraacetic acid
ERBB2	erb-b2 receptor tyrosine kinase 2
ERCC2	excision repair cross-complementation group 2
ESCAT	ESMO Scale for Clinical Actionability of molecular Targets
ESMO	European Society for Medical Oncology
EV + P	enfortumab vedotin plus pembrolizumab
FDA	Food and Drug Administration
FGFR2/3	fibroblast growth factor receptor 2/3
GC	gemcitabine-cisplatin
HER2	human epidermal growth factor receptor 2
HR	hazard ratio
ICI	immune checkpoint inhibitor
IHC	immunohistochemistry
ISLB	International Society of Liquid Biopsy
ITT	intention-to-treat
LB-RECIST	Liquid Biopsy Response Evaluation Criteria in Solid Tumors
LOB	limit of blank
LOD	limit of detection
MIBC	muscle-invasive bladder cancer
mOS	median overall survival
MRD	molecular residual disease
mUC	metastatic urothelial carcinoma
NACT	neoadjuvant chemotherapy
NGS	next-generation sequencing
NMIBC	non-muscle-invasive bladder cancer
OS	overall survival
PCR	polymerase chain reaction
PD-L1	programmed death-ligand 1
PFS	progression-free survival
RCT	randomized controlled trial
RECIST	Response Evaluation Criteria in Solid Tumors
RT-PCR	reverse transcription polymerase chain reaction
T-DXd	trastuzumab deruxtecan
UC	urothelial carcinoma
utDNA	urinary tumor DNA
VAF	variant allele frequency
WES	whole exome sequencing
WGS	whole-genome sequencing
