# Supplementary material for: Dynamic Precision Oncology for Real-Time Molecular Monitoring and Management in Urothelial Carcinoma
Source: Int J Mol Sci. 2026 Apr 13;27(8):3474. doi: 10.3390/ijms27083474 (PMC13116755; doi:10.3390/ijms27083474)

Figure S1. Dynamic liquid biopsy workflow for ctDNA-guided management of urothelial carcinoma. Pre-analytical steps include patient selection and consent, blood collection into cell-free DNA stabilizing tubes, timely transport, and standardized plasma processing to preserve integrity of cfDNA. Analytical steps comprise cfDNA extraction, library preparation, and sequencing by next-generation sequencing or digital PCR, followed by bioinformatics analysis including CHIP filtering using paired leukocyte DNA. Post-analytical steps consist of structured reporting in the electronic medical record of the patient and multidisciplinary molecular tumor board review to inform treatment and surveillance decision-making

CHIP, clonal hematopoiesis of indeterminate potential; PCR, polymerase chain reaction; NGS, next-generation sequencing; EMR, electronic medical record. Created in BioRender. Kwon, W. (2025) https://BioRender.com/undefined

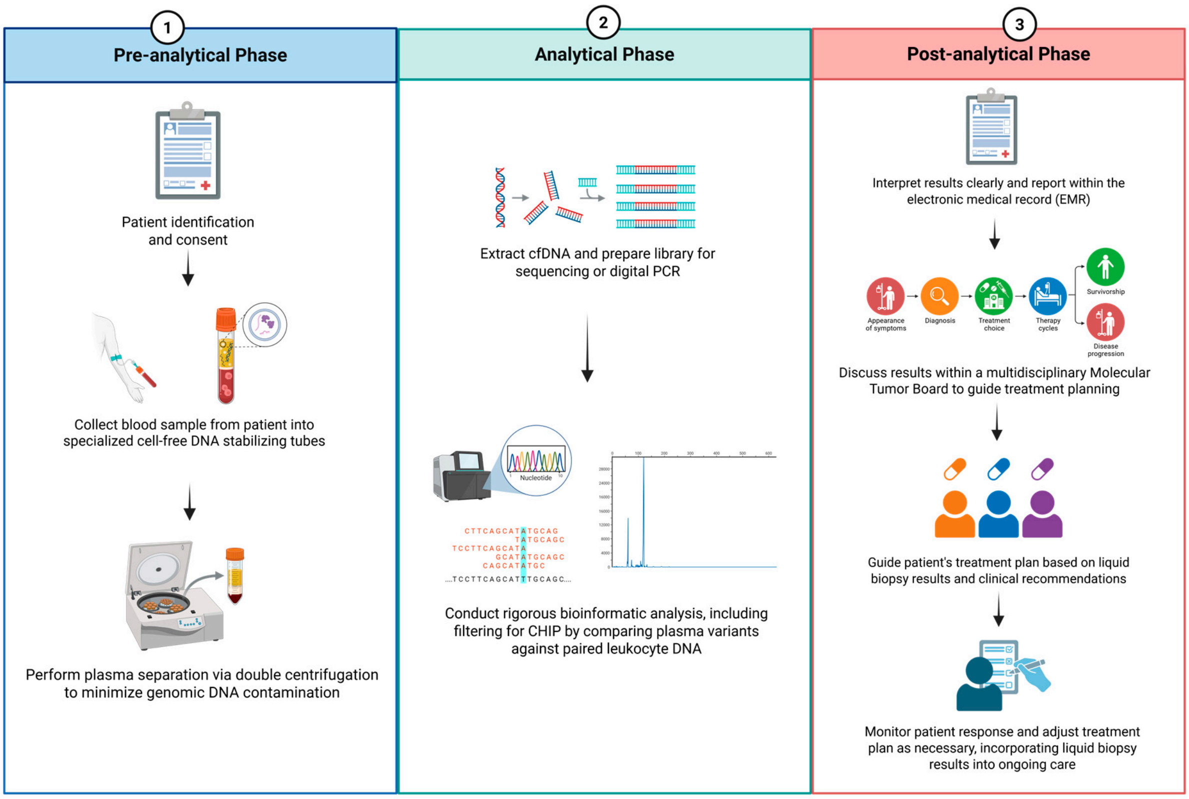

Supplement: Supplementary file 1 [file ijms-27-03474-s001.zip › ijms-4183434-supplementary.pdf]
